# The influence of contextual factors on healthcare quality improvement initiatives: a realist review

**DOI:** 10.1186/s13643-020-01344-3

**Published:** 2020-04-26

**Authors:** Emma Coles, Julie Anderson, Margaret Maxwell, Fiona M. Harris, Nicola M. Gray, Gill Milner, Stephen MacGillivray

**Affiliations:** 1 Nursing Midwifery & Allied Health Professions Research Unit (NMAHP-RU), University of Stirling, Scion House, University of Stirling Innovation Park, Stirling, FK9 4NF UK; 2 Scottish Improvement Science Collaborating Centre (SISCC), School of Nursing and Health Sciences, University of Dundee, 11 Airlie Place, Dundee, DD1 4HJ UK; 3 School of Nursing and Health Sciences, University of Dundee, 11 Airlie Place, Dundee, DD1 4HJ UK

**Keywords:** Realist review, Realist synthesis, Context, Quality improvement, Health improvement, Implementation, Healthcare, Evidence-based practice, Knowledge translation

## Abstract

**Background:**

Recognising the influence of context and the context-sensitive nature of quality improvement (QI) interventions is crucial to implementing effective improvements and successfully replicating them in new settings, yet context is still poorly understood. To address this challenge, it is necessary to capture generalisable knowledge, first to understand which aspects of context are most important to QI and why, and secondly, to explore how these factors can be managed to support healthcare improvement, in terms of implementing successful improvement initiatives, achieving sustainability and scaling interventions. The research question was how and why does context influence quality improvement initiatives in healthcare?

**Methods:**

A realist review explored the contextual conditions that influence healthcare improvement. Realist methodology integrates theoretical understanding and stakeholder input with empirical research findings. The review aimed to identify and understand the role of context during the improvement cycle, i.e. planning, implementation, sustainability and transferability; and distil new knowledge to inform the design and development of context-sensitive QI initiatives. We developed a preliminary theory of the influence of context to arrive at a conceptual and theoretical framework.

**Results:**

Thirty-five studies were included in the review, demonstrating the interaction of key contextual factors across healthcare system levels during the improvement cycle. An evidence-based explanatory theoretical model is proposed to illustrate the interaction between contextual factors, system levels (macro, meso, micro) and the stages of the improvement journey. Findings indicate that the consideration of these contextual factors would enhance the design and delivery of improvement initiatives, across a range of improvement settings.

**Conclusions:**

This is the first realist review of context in QI and contributes to a deeper understanding of how context influences quality improvement initiatives. The distillation of key contextual factors offers the potential to inform the design and development of context-sensitive interventions to enhance improvement initiatives and address the challenge of spread and sustainability. Future research should explore the application of our conceptual model to enhance improvement-planning processes.

**Systematic review registration:**

PROSPERO CRD42017062135

## Contributions to the literature

- Spreading and sustaining quality improvement (QI) initiatives in healthcare is a recognised challenge; this research highlights the influence of contextual factors on these efforts.
- Although the evidence base around context in QI is increasing, there remains limited knowledge and guidance about which contextual factors are most influential. This review explores how, when and for whom context impacts during the improvement journey across healthcare system levels.
- This is the first realist review of context in QI. The realist approach incorporates theory, research evidence and practical knowledge to facilitate the exploration of the multi-level, multi-faceted nature of context, within complex healthcare settings.

## Background

Improving health and wellbeing outcomes is a key focus of public sector organisations. Over the last decade, there has been significant effort to utilise quality improvement (QI) within healthcare, as a means of delivering evidence-based care, improving mechanisms of care and clinical outcomes. Quality improvement is the purposive, systematic application of specific methods to improve service configuration or delivery, in order to achieve positive change. Key features are identified as ‘the combination of a ‘change’ (improvement) and a ‘method’ (an approach with appropriate tools), while paying attention to the context, in order to achieve better outcomes’ [1]. Some definitions go further, i.e. ‘healthcare improvement’, which incorporates changes leading to ‘better patient outcomes (health), better system performance (care) and better professional development’ [2]. Within the literature, there is also a distinction made between improvement interventions (for example, bundles or checklists) and the ‘doing of improvement’, the QI approaches and methods used to implement these interventions [3].

Despite such explicit definitions and approaches, QI results are often mixed, unpredictable or demonstrate limited impact [4], suggesting that translating evidence into practice and implementing improvement initiatives to achieve effective change is not straightforward. One of the key challenges in healthcare improvement is that what works in one setting does not always readily transfer to other settings [5, 6]. This suggests that many improvements are context-sensitive or even context-dependent [7], and failure to replicate the impact of previously successful improvement efforts in new settings is often attributed to the ‘problem’ of context [8]. Context is a diverse range of conditions that influences the implementation, effectiveness and spread and sustainability of QI initiatives [9–11], hence the need to build context into the systematic approach of QI.

### How is context understood?

The definition of context in relation to QI has evolved over time [12]. At its most simplistic, context can be defined as ‘all factors that are not part of a quality improvement intervention itself’ [11], i.e. ‘the set of characteristics and circumstances or unique factors that surround a particular implementation effort’ [9]; or anything not directly part of the QI process or intervention [10]. It has also been described as the underlying systems, culture and circumstances of the environment in which an intervention is implemented [7].

Within the literature, contextual factors are frequently conceptualised as either barriers or facilitators; however, this may be too simplistic [12]. The SQUIRE 2.0 publication guidelines for quality improvement studies in healthcare [13] recognises context as one of the fundamental reporting items. These guidelines reflect the complex nature of context as the ‘key features of the environment in which the work is immersed and which are interpreted as meaningful to the success, failure, and unexpected consequences of the intervention(s), as well as the relationship of these to stakeholders (e.g. the improvement team, clinicians, patients…)’ [13].

### Why is context important to quality improvement?

Healthcare systems are complex and dynamic, and as such, their contextual interactions are not static. Throughout the improvement journey, different aspects of context can assume more or less importance (exerting more or less influence), depending on the type of intervention being implemented, its infancy or maturity, stage of implementation, system level at which it is targeted, and, in the case of multi-component interventions, specific components [14]. Contextual ‘confounders’ that act as barriers to improvement in one setting may be facilitators in other settings; such confounders often represent ‘typical’ healthcare conditions [15].

Until relatively recently, improvement and implementation science research paid little attention to context, instead focusing on outcomes, effectiveness and impact. Frequently within research studies, contextual attributes were either not acknowledged, under-reported [16–18] or viewed as confounding variables to be controlled for or eliminated, despite being a key determinant of implementation success or failure and spread/sustainability [19].

Given that improvement is an inherently context-dependent social process, taking place in real-world clinical settings [20], the influence of context is highly significant, and so stripping out contextual factors from improvement research limits the usefulness and generalisability of findings [21]. Research studies that factor out context are not always able to articulate the crucial ‘how’ and ‘why’ around the success or failure of QI projects within complex healthcare systems, and as such, are not able to predict whether improvement efforts will easily transfer to other settings.

In recent years however, there has been a growing recognition of the influence of context on healthcare improvement efforts [22, 23], with acknowledgement that QI interventions cannot be understood outwith their implementation settings [24]. The variability within the body of literature in this area [9, 16, 17, 25] suggests that further work is required to unpack the role of context, and explore which characteristics of context matter, and how, why, when and for whom they matter.

### Review purpose

This realist review was designed to further theoretical understanding of which aspects of context are important and why, and how these factors can be addressed and managed to support healthcare improvement efforts. The research question was how and why does context influence quality improvement initiatives in healthcare? More specifically, the review aimed to (i) identify contextual factors that influence the implementation, effectiveness, sustainability and transferability of QI initiatives in healthcare; (ii) provide a theoretical explanation of how, why, when and for whom these factors are important; and (iii) provide stakeholders (improvement practitioners, clinicians and policymakers) with a practical, up-to-date evidence base relating to the influence of context.

### The realist approach

Incorporating theory, research evidence and practical knowledge, realist inquiry is ideal for understanding the issues surrounding implementation in complex healthcare settings. This theory-driven interpretive approach seeks to explain the causes of intervention outcomes and patterns of outcomes and effects, by evaluating knowledge and data from a range of sources [26]. Based on the realist assumption that all interventions and programmes have underlying hypotheses outlining how they are assumed to work, and the factors that might cause change, realist ‘programme theories’ are expressed in terms of context, mechanism and outcome (CMO) configurations [26, 27].

The starting point for the notion of context in realist review is the four contextual ‘layers’ defined by Pawson et al [27]: individuals, interpersonal relations, institution and infrastructure. These realist conceptualisations of context refer to any characteristic of the individual capacities of key actors; the interpersonal relationships between stakeholders; the institutional setting; and the wider societal, economic and cultural infrastructure. This appreciation of context and complexity is significant: the realist approach acknowledges that because interventions are governed and conditioned by the contexts that they are embedded in, there is an inherent challenge with regard to transferability to other settings [26]. This is because factors within particular contexts enable certain mechanisms to trigger outcomes and therefore interventions cannot simply be transferred from one context to another and be expected to achieve the same results [28, 29]. However, realist understandings of ‘what works, for whom and in what settings’ are portable and can generate transferable, generalisable lessons [30].

## Methods

**Fig. 1:**
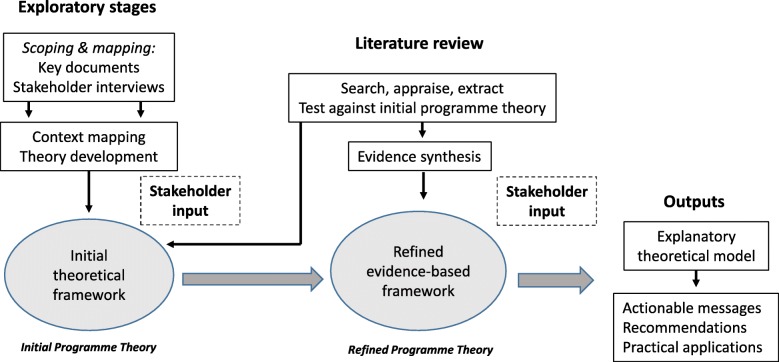
Overview of the realist review process

We followed a template adapted from Pawson [26]: (1) define scope of review and develop theoretical framework (exploratory background literature searching, stakeholder consultation, theory development); (2) theory-driven purposive search for evidence; (3) appraise evidence and extract data; (4) synthesize data and draw conclusions; (5) disseminate findings. The realist review process is iterative and non-linear, with considerable overlap between stages and work on different steps undertaken simultaneously (Fig. 1). The review was conducted and reported in accordance with the Realist And Meta-narrative Evidence Syntheses: Evolving Standards (RAMESES) guidance and publication standards [31, 32]. No changes were made to the published review protocol [33].

### Exploratory stage: defining the scope of the review and theory development

The objective of the first stage was to understand the scope of the review and develop the programme theory. This involved a number of interconnected iterative processes: scoping (exploratory background literature searching); mapping (defining key themes and concepts, conceptualising context); and consultation with stakeholders and experts. All aspects of this preliminary work informed the programme theory development. A multi-disciplinary advisory group of academics and improvement practitioners was set up to oversee the review, monitor progress, develop consensus and contribute to theory development and interpretation of findings.

In the initial exploratory stage, the review team conducted background searches and consulted with key stakeholders from policy, practice and academia, to map the terrain, refine the research question, and clarify the focus and breadth of the review. This scoping exercise included the identification and scrutiny of key publications examining the role of context in healthcare quality improvement [10, 11, 14, 16, 23, 34, 35]. This approach enabled the review team to ‘get a feel’ for the topic whilst simultaneously gaining a deeper understanding of the research problem.

#### Stakeholder involvement

Involving stakeholders in realist research provides a range of additional perspectives and an ‘expert framing’ of the issues that could contribute to the developing programme theory [27]. Initial stakeholder consultation took the form of telephone interviews with 15 informants in the field of healthcare improvement, lasting between 30–45 min. All except one participant was located in Scotland; the other contributor was based in England. Participants held a range of roles within improvement, and several held dual posts spanning both the NHS and academia. The participants provided representation from the three system levels: macro (policy), meso (national organisation implementation and support roles) and micro (local implementation remit). Accordingly, their viewpoints reflected the various ways in which the different system levels exerted influence on their improvement-related activities. Participants were asked for their views on the role and influence of context in the implementation of QI initiatives. Fifteen interviews were carried out until saturation was reached. The inclusion of stakeholder views and thinking around the impact of context in improvement during the exploratory stage provided rich contextual information, and key themes emerged to support the development of the initial programme theory.

**Fig. 2:**
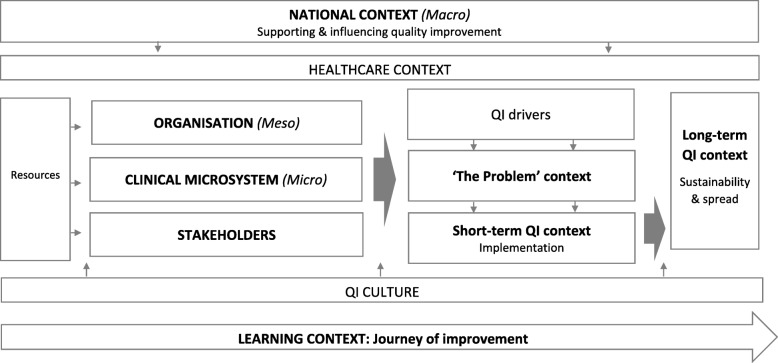
Healthcare QI context map

Findings from the exploratory search and insights from stakeholders and experts generated a number of potentially relevant contexts. As part of the realist theory development, the key contexts were mapped to the landscape of healthcare QI within Scotland (using NHS Scotland’s whole-systems approach to quality improvement as the starting point for theory creation) to produce a provisional ‘context map’ (Fig. 2).

The provisional context map formed the basis of further stakeholder engagement. Mapping out the quality improvement landscape within Scotland to represent the emergent theory enabled stakeholders to engage in the exploration of potential contexts, mechanisms and outcomes across macro, meso and micro system levels. This process advanced the initial theory into a generalisable programme theory, applicable to a range of improvement settings.

#### Developing a generalisable theory of context in QI

**Fig. 3:**
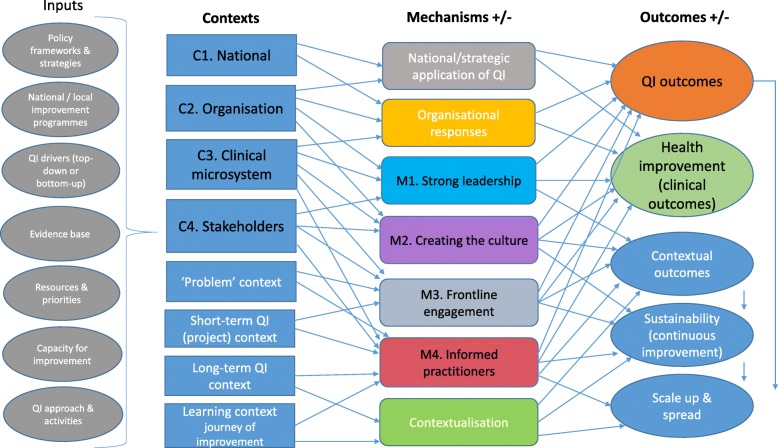
Realist framework based on programme theory

Hypothesizing how improvement activity within and between the different contexts was likely to play out in terms of the associated mechanisms and outcomes; we developed a realist programme theory, expanding the provisional context map. The programme theory was our conceptualisation of the healthcare improvement landscape, and the role and influence of contextual factors, representing the interactions between multiple components and multiple levels within a complex system, and illustrating context, mechanism and outcome relationships and the patterns of outcomes and effects. The programme theory, which formed the theoretical framework for the subsequent stages of the review, is summarised in Fig. 3 and Table 1.

**Table 1:** Realist programme theory summary

C. Context	I. Inputs/resources	M. Key mechanisms	M. Other mechanisms	O. Outcomes
**C1 National policy context** National drivers National QI support Continuous QI culture	Policy frameworks and strategies National improvement programmes Resources	M Consistently supporting and encouraging improvement (coherent message) M National/strategic application of QI	Continuous focus on QI Fit between national agenda and local priorities	QI-related outcomes -sustainability -scale and spread Health improvement outcomes Practitioner (clinical/frontline) outcomes Contextual outcomes Negative/unanticipated outcomes
**C2 Organisation** Leadership Culture Systems & processes	Policy frameworks National improvement programmes Local improvement programmes Resources In-house QI support/coaching	M Organisational responses **M1 Strong leadership** **M2 ‘Creating the culture’**	Top-down: Strategic/administrative engagement and participation Multi-disciplinary collaboration	
**C3 Clinical microsystem (Frontline)** Context of change	Developing capacity and capability Evidence for change + Intervention Diagnostic work (contextualisation) QI approach	**M2 ‘Creating the culture’** **M3 Frontline engagement** M Developing capacity for improvement M Co-creation/co-production M Ownership **M4 Informed practitioners**	Bottom-up: Buy-in/commitment Ownership	
**C4 STAKEHOLDERS** Context of individual change	QI approach Evidence for change + Intervention	**M3 Frontline engagement** M Co-creation/co-production **M4 Informed practitioners** M Common understanding and language M Buy-in/commitment	Passion vs resistance ‘Having the conversation’ ‘Permission’	
**‘The Problem’ context**	Solution/intervention (the IDEA; the ‘change’)	**M3 Frontline engagement** M Awareness M Willingness	‘Permission’	
**Short-term QI [project] context** Implementation context	QI approach/activity Intervention Introducing/testing change	M ‘Will— ideas—execution’ M Aligning	Ownership	
**Long-term QI context** Post-intervention context	Intervention QI approach	M Contextualisation	Rate/pace of change Long-term embedding	
**Learning context (‘journey of improvement’)**	QI approach QI mentoring	**M4 Informed practitioners** M Choice M Starting the journey	Sharing ‘what works’ Relationship with failure	

### Purposive, theory-driven evidence searching

#### Realist searching processes

The realist approach aims to retrieve sufficient evidence to answer the research question and achieve theoretical saturation, as opposed to a fully comprehensive search [26]. Review evidence from a range of sources was drawn from iterative, broad-brush exploratory searches; these preliminary searches identified a large number of potentially relevant articles that were appraised for inclusion. Further evidence was located via a broad range of methods: electronic database searches, using index terms and free text; reference scanning; citation tracking; searching websites of relevant peer-reviewed journals for QI reporting; electronic alerts, i.e. from Google Scholar, databases, relevant journals; and grey literature searches, including Google searching. Stakeholders with knowledge and experience in delivering QI initiatives and education, from a wide range of organisations, including the National Health Service (NHS), were approached to support and contribute to the search strategy.

#### Search strategy and eligibility criteria

The search strategy was purposefully broad and driven by the programme theory as it developed and was refined through the course of the review. At the review outset, a number of broad-brush exploratory scoping searches were carried out, yielding a large number of full-text documents, including reviews. During programme theory development, further, mostly informal, searches were conducted iteratively (reflecting the current thinking about the programme theory at each point as it evolved) and additional full-text documents were retrieved and stored, in anticipation of the ensuing stages of the review.

These preliminary searches produced a large number of potentially relevant articles. Assessment of these existing documents was carried out prior to further searching; six of these were included in the final synthesis. Once these documents were screened, selected and appraised, further searches were carried out, building on the evidence generated by the preliminary searches, in order to find additional pertinent evidence to further test and refine the programme theory. Although this included electronic database searching, the majority of studies were located by other means, including searches of relevant journals, electronic alerts, and via informal methods including from personal contacts (project team members or stakeholders) or by ‘serendipitous discovery’. Hence, this second search phase identified some additional articles (e.g. process evaluations and qualitative research studies), to test and refine the evolving programme theory; however, the aim was not to be fully comprehensive, but to identify relevant literature sufficient to enable the role of context to be explored.

The eligibility criteria was set to include empirical research studies of QI initiatives, in primary or secondary healthcare settings, published in English during the previous 10 years, based in developed, industrialised countries. Quality improvement in healthcare is a relatively new area, [1, 8] furthered by the work of the Institute for Healthcare Improvement in the USA [1]. Hence, it was decided to focus on higher-income countries, due to the emphasis placed on improving the quality of healthcare systems in these countries where the use of QI approaches is established and more ‘mature’. Limiting inclusion criteria to the previous 10 years was a similarly pragmatic decision, to restrict the review to relatively recent publications whilst at the same time capturing sufficient evidence within a manageable data set. Further, findings from the background search suggested that the evidence base prior to this point would be unreliable, given that during the first decade of the twenty-first century, QI was considered a relatively new and developing field for health services research [8], and as a result, contextual issues would be less likely to be explicitly acknowledged or reported in older studies.

### Selection and appraisal of documents

The realist selection and appraisal process differs from a traditional systematic review. Assessing whether research is fit for purpose according to relevance and rigour is the realist alternative to quality appraisal in a systematic review. Decisions about rigour and relevance were made on the basis of potential contribution(s) of the study either as a whole or a section could make to the review.

#### Relevance

In a realist review, the unit(s) of assessment is not each included study itself or the intervention it describes, but any sections of the study that are relevant to underlying theory and context-mechanism-outcome evidence. Within a document, different kinds of evidence may be relevant to different aspects of the review or the programme theory [32]. Selection of documents for inclusion was based on whether the document as a whole contained any type of evidence, from any part of the study (not just the results or findings), that could contribute to the development, testing, corroboration and/or refinement of any aspect of the programme theory. Decisions were based on, for example whether papers provided any contextual data, data relating to potential mechanisms, identifiable outcomes or CMO examples (either implicit or explicitly author-identified). The various types of evidence (e.g. participant quotes) were recorded and aligned with appropriate aspect(s) of the programme theory (e.g. context, mechanisms or outcomes).

#### Rigour

Documents were then assessed for methodological rigour (whether the methods used to generate the relevant data were credible, plausible and trustworthy), whilst bearing in mind that even methodologically weak studies that would be otherwise excluded by a traditional systematic review may contain potentially valuable ‘nuggets’ of understanding [36]. Assessment of rigour therefore focused the extent to which studies provided a detailed description of methods and the level of generalisability of findings. The methodological limitations of any studies included in the review or any particular issues around data quality were noted and considered during the analysis and synthesis.

### Data extraction, analysis and synthesis processes

Data extraction, analysis and synthesis was an iterative process beginning with familiarisation and understanding of each study. Each included study was read and re-read, initially for familiarisation and then to assess its relevance to the evidence relating to underlying theory and relevance to the research questions. Within each document, relevant passages containing key evidence were highlighted, annotated and coded to identify contexts, mechanisms, outcomes and CMO configurations. Documents were also examined to capture explanatory accounts, themes, concepts and any other relevant data that might contribute to theory refinement.

‘Bespoke’ data extraction forms were created to capture information from each article on contextual factors, mechanisms and outcomes, along with additional data on QI methodology and implementation. A data extraction template and sample extraction table is available on request from the corresponding author. EC conducted the full data extraction; JA independently reviewed each study; and NG and GM checked a 10% sample for credibility of theory development and refinement and reliability of extraction. Using processes of abstraction and conceptualisation, the reviewers compared and contrasted the evidence, looking for patterns of CMOs across the data that were able to support, contradict or inform the programme theory. Recurring themes were also identified and used to guide the rest of the review process as data extraction and analysis progressed.

## Results

### Included studies

Thirty-five studies published between 2010 and 2018 were identified for inclusion (Table 2). The majority (*n* = 33) were peer-reviewed articles; two were reports. Most studies were from the UK (*n* = 16) and USA (*n* = 8), with the remaining studies from Europe (Norway, Holland, Republic of Ireland), Canada and Australia. Although a variety of study designs were represented, studies were predominantly qualitative, including two realist evaluations. Five were mixed-methods, and two were embedded in wider studies. One study used a longitudinal design, and two involved secondary analysis. Thirteen studies specifically aimed to explore the influence of context or contextual factors. Others addressed contextual issues indirectly in the form of organisational culture, barriers and facilitators to implementation, or improvement capacity and capability. Nineteen studies used a guiding theoretical model or framework, most commonly PARIHS (*n* = 5) and MUSIQ (*n* = 4).

**Table 2:** Included studies

Author, year, country	Clinical area improvement interventions occurred	Improvement aim	Care or quality gap; deficiency that intervention aims to address	Context description/features
Armstrong et al. 2016, UK [37]	Primary care practices	Improve the quality of the chronic kidney disease (CKD), register and implement the CKD bundles and introduce self-management tools	Improve quality of the CKD register.	Unique features of primary care setting: prioritisation, lack of mechanisms to mandate engagement, working relationships (locus of power—nurses were implementers but GPs/Practice Managers needed to authorise), alignment with financial (and other) incentives; the degree of fit between the intervention and the context in which it was being implemented as the most influential interrelationship.
Benning et al. 2011, UK [38]	Designated clinical areas in 4 UK hospitals	Improve reliability of specific frontline care processes in designated clinical specialities and promotion of organisational and cultural change	Improve reliability of care processes across different clinical sites within hospitals and develop safety culture and good leadership to enable organisational management of problems and risk.	Organisational climate. Gap between strategic level and frontline.
Boaz et al. 2016, UK [39]	Intensive care units and lung cancer pathways	Implement improvement priorities identified through a participatory/co-design process	Intensive care units prioritised improvements in enhancing basic care, reducing noise and sleep deprivation, communication, patient-doctor communication on ward rounds, transition to the ward: ‘lost in translation’, hallucinations, ventilation and individualised care. Lung cancer pathways prioritised improvements in pillows, personal items, information, privacy, diagnosis-giving, support and information.	Patient and carers experience/working alongside staff. This type of engagement focused on ‘smaller scale’ improvement—rather than the current focus of large-scale change and identified the benefits of this approach to the broader cultural challenges around the acceptability of change.
Carney 2011, Republic of Ireland [40]	Clinical and non-clinical heads of departments within hospitals	n/a—study was an exploration of the pivotal role of head of department/directorate to healthcare management and its influence on healthcare planning and quality healthcare delivery.	n/a	Organisational culture. Leadership.
Curry et al. 2018, USA [41]	Hospital-wide leadership and organisational culture	Outcomes were change in culture, uptake of five strategies associated with lower risk-standardised mortality rates (RSMR).	Hospital organisational culture affects patient outcomes including lower risk-standardised mortality rates (RSMRs) for patients with acute myocardial infarction; little is known about whether and how culture can be positively influenced.	Organisational culture as a contextual factor that can accelerate learning and improvement; impacts on adoption of EBP.
Darley et al. 2018, UK [42]	Maternity services	n/a—study was an exploration of the utilisation of an improvement capability assessment tool.	Variations in service performance and quality improvement.	Organisational context; interactions between organisational performance and improvement capability; division of intervention and context is arguably somewhat artificial—the two interact in multiple, complex and dynamic ways.
Dixon-Woods et al. 2013, UK [43]	Adult intensive care units	Reducing central line bloodstream infections; improvements in patient safety and reduce 30 day mortality.	Decline effect and failure to outperform secular trend seen in replication of QI/PSP initiatives. Healthcare-acquired infections and central line catheter-related blood stream infections—reduce morbidity and mortality associated with these infections.	Broad: national to local, influence of inner and outer, context as culture, context as implementation climate. Outer context—national infection control policies—top down and punitive. Local context—experience of QI initiatives, data collection capability, feedback systems, local leaders to develop consensus and coalition. Improved understanding of contexts of implementation may reduce risks of decline effects and add value beyond secular trends.
Dückers et al. 2011, Holland [44]	Across hospital organisations	Stimulate the development of quality management systems and the spread of methods to improve patient safety and logistics	Address the lagging development of quality management systems optimisation of healthcare delivery through organisational-wide diffusion and quality improvement programmes.	Macro: stimulating physicians to join quality-improvement initiatives but also by adopting the organisational strategy for sustainability and dissemination, national performance measures and policy. Meso: leadership and performance management—align vision and quality, create feedback loops between layers and internal programme structure. Micro: QI training from external experts. System changes affect the context factors in the theory of organisational readiness: organisational culture, policies and procedures, past experience, organisational resources, and organisational structure. These factors are utilised to manage spread and sustainability.
Edward et al. 2017, Australia [45]	Operating rooms and recovery/post-surgical care wards	Reduce the incidence of inadvertent perioperative hypothermia	Slow process of translating research; need for effective translational research models to ensure patient care quality and safety are not compromised. Strong evidence that mild intraoperative hypothermia quadruples the risk of surgical site infection, doubles the risk of perioperative myocardial events and significantly increases surgical blood loss.	Stakeholders. Frontline: clinicians, teams, collaboration. Learning systems.
Flynn and Hartfield 2016, Canada [46]	Paediatric Intensive Care Unit	Improve hand hygiene practice within the paediatric intensive care unit	Need to understand barriers and facilitators around implementing initiatives in complex systems. Many quality issues and adverse events in healthcare are preventable. Poor quality and adverse events are costly to healthcare systems. Infections are preventable harm.	Individual, unit and organisational; QMF as a whole system changes mechanism. Leadership—different system levels.Organisational culture—general interest from leading physician in QI and strong working relationships between physicians and nurses. Resources (or lack of)—personnel and QI knowledge. Complex social interventions—a variety of contexts across multiple levels of the healthcare system: patient, healthcare provider, multidisciplinary team, institution and local and national healthcare system levels.
Gagliardi et al. 2014, Canada [47]	Colon cancer screening, prostate cancer diagnosis, pancreatic cancer treatment services	Three areas of clinical priority identified by the cancer agency—increase update of colorectal screening, reduce overuse of prostate cancer screening and reduce mortality associated with pancreatic cancer.	Collaboration among researchers (clinician, non-clinician) and decision makers (managers, policy-makers, clinicians), referred to as integrated knowledge translation (IKT), enhances the relevance/use of research, leading to improved decision-making, policies, practice, and health care outcomes. But IKT is not widely practiced due to numerous challenges. Focus was the improvement of clinical areas identified by provincial cancer agency.	Culture receptive to change, leadership support, feedback to staff (PARIHS). Organisation: culture, leadership, capacity (infrastructure, political, economic, social). Individual: professional role, involvement, personal characteristics. Contextual factors at the individual (knowledge, beliefs, motivation) and organisational (culture, leadership, capacity) levels.
Gingold et al. 2016, USA [48]	Paediatric primary care	Increase the uptake of childhood immunisations.	Routine childhood immunisation can prevent morbidity and mortality. Uneven adherence to immunisation guidelines leaves some communities vulnerable to outbreaks of vaccine-preventable diseases.	Data infrastructure, management structure, interpersonal interactions, beliefs and behaviours.
Gjestsen et al. 2017, Norway [49]	Home-based care services	National programme established to develop and implement assistive living technologies is integrated in primary care services by 2020.	Assisted living technologies—help monitor and treat degenerative and chronic diseases that follow an ageing society through the use of sensors, alarms and reminders and could be used to prevent hospitalisations by providing early warnings of exacerbation events or deterioration.	MUSIQ—microsystem, QI team, healthcare system macro (external, policy), meso (organisation factors), micro. Context factors interdependent and mutually reinforcing. Acknowledges the organisational, social, political and policy context.
Green et al. 2017, UK [50]	Acute medical hospital wards	COPD bundle aims to improve the quality and consistency of the care received by patients, and to reduce variations in care processes and clinical outcomes. Diabetic foot care—improve screening and management of in-patient diabetic foot complications based on current best practice guidelines.	Challenge of consistent implementation of clinical guidelines: implementation of care bundles developed from guidelines to deliver evidence-based changes COPD is associated with significant morbidity and mortality—following hospitalisation, consistency in care during admission, discharge and follow-up care has been shown to reduce readmissions and improve clinical outcomes. Timely identification and management of diabetic foot can prevent significant complications (lower limb amputation) and reduce associated morbidity, improving clinical outcomes.	Organisation and stakeholder/practitioner level. Implementation climate.
Grooms et al. 2016, USA [51]	Neonatal Intensive Care Units	Focus on clinical and value improvement with specific focus on the standardisation of processes and understanding context.	Need to systematically address role of context and how to make local context more supportive. Identify gaps and design improvements in QI context to ensure QI initiative is successful. Improve clinical and value outcomes and standardise processes within neonatal units.	Organisation; microsystem; data infrastructure.
Hamilton et al. 2014, Canada [52]	Surgical units in tertiary and secondary hospitals in Saskatchewan	Implement RTC in all surgical units in tertiary and secondary hospitals in Saskatchewan.	Consistent approach to QI for nurses is needed to avoid isolated pockets of excellence and ensure projects are aligned and not competing for attention. Enables staff to identify areas for continuous improvement and aims to increase the amount of time nursing staff have to spend with patients.	Organising for quality domains: educational, structural, cultural, political, physical, technical. Highlights the importance of understanding existing context when considering QI implementation and the limitations of mandated top-down imposed QI initiatives.
Harvey et al. 2018, UK [53]	Secondary care settings including specialist children’s services and specialist diabetes clinic	Increase the uptake of IPT to 12% of adults and 33% of children < 12 years old.	Accelerating innovative technology uptake in the NHS; facilitation role of national agencies. Insulin pump therapy is a clinically and cost effective treatment of people with Type 1 Diabetes where multiple daily injections have failed.	Leadership support; culture; past experience of innovation and change; structure, systems and processes; organisational priorities; policy drivers; incentives and mandates; inter-organisational networks and relationships. Factors related to the organisation and delivery of healthcare: politics and culture at a local level, alongside organisational and system level issues related to funding and commissioning new technologies.
Hovlid and Bukve 2014, Norway [54]	Wards and departments involved in the clinical pathways delivering elective surgery	Redesign the clinical pathway for elective surgery to reduce cancellations and sustain system improvements	Influence of contextual factors on QI processes and outcomes. Cancellation of scheduled surgery is a quality of care problem.	Healthcare system; clinical system. Follows Øvretveit view of interactions of contextual factors with each other and with implementation process.
Kaplan et al. 2016, USA [55]	Maternity hospitals	Antenatal corticosteroids (ANCS) to reduce preterm birth complications.	Preterm birth is a leading cause of neonatal morbidity and mortality—antenatal corticosteroids can reduce the complications of preterm birth but many hospitals do not have the right processes and conditions for reliable implementation of ANCS.	Inner and outer settings. High reliability culture, culture and physician leadership. ‘General elements of context, evidence and facilitation are also important in sustaining evidence delivery at high levels’. Contextual influences on the sustainability of improvements.
Krein et al. 2010, USA [56]	Intensive care units	Reduce central-line bloodstream infections.	Hospital patient safety; infection control. Prevention of central line-associated bloodstream infections (CLABSI).	Structure (leadership, culture, resources, co-ordination); people; champions. ‘We also need to better understand when, how or even which practices and implementation strategies might work given the organizational context’. Which organisations might be more receptive to collaboratives and externally-facilitated efforts.
Manley et al. 2017, UK [57]	Wide range of inpatient settings within hospitals—maternity departments, A&E, ambulatory care and specialist care wards	Implementation of safety huddles and other QI tools, Teamwork Safety Climate Survey, and action learning for the facilitators supporting frontline teams.	Patient safety collaboration to embed a safety culture, grow leadership and quality improvement capability.	Culture; interconnections within the organisation between the frontline teams and leadership.
McCullough et al. 2015, USA [58]	Pharmacy-run anticoagulation clinics	Implementation of an evidence-based anticoagulation treatment algorithm as part of the regional Anticoagulation Clinical Improvement Initiative; implement processes of care to improve follow-up actions and reduce loss to follow-up.	Strength of contextual elements and their effects; interactions between contextual elements. Improve anticoagulation care and reduce rates of patient complications.	Dynamic, multivalent and highly variable in organisational life. Contextual elements multidimensional: e.g. evidence, leadership, teamwork, communication. Ranked as strong, moderate or weak in relation to initiative. Interrelationships among different contextual elements can act as barriers to uptake at some sites and as facilitators at others—predictor of uptake of intervention.
Meehan et al. 2015, USA [59]	Skilled nursing facilities (SNF) (UK equivalent of nursing homes)	Reduce preventable hospital readmissions through improving the identification, evaluation and management of acute changes in the conditions of SNF residents.	Decreasing preventable hospital readmissions from SNFs—in 2011 25% of Medicare beneficiaries discharged from hospital to a SNF had at least one readmission within a year.	Institution-specific (e.g. culture, leadership); organisations as complex adaptive systems.
NIHR CLAHRC Greater Manchester 2018, UK [60]	Hospital-based wards	Improve the identification and management of acute kidney injury.	AKI is a preventable clinical syndrome; need to achieve better identification management in hospital care.	National, regional, local (organisational context).
Papoutsi et al. 2018, UK [61]	Acute Medical Units or equivalent	Aim to reduce harm and increase assessment reliability for older people admitted acutely to hospital, through the introduction of a checklist to increase completion of key clinical admission assessments and improve communication.	Older patients with multiple co-morbidities suffer from disproportionate levels of harm in their care due to insufficient attention to frailty in non-specialist settings.	System of pre-existing patterns of working, communication and sharing responsibility.
Phung et al. 2016, UK [62]	Emergency care pathways for Ambulance Service care bundles for acute myocardial infarction and stroke	Increase the reliability of delivering the AMI (> 70%) and stroke (> 90%) care bundles.	Ambulance services are an important component of care pathways for emergencies and will influence morbidity and mortality outcomes.	Organisational culture, clinical leadership, culture of innovation. Leadership and organisational culture also contextual factors for clinical governance.
Power et al. 2016, UK [63]	Range of primary and secondary care settings	Develop a shared national, regional and locally aligned safety focus on 4 harms, establish measurement system to capture harm-free care and deliver improved outcomes.	Promote an innovative approach to patient-centred harm-free care to address the challenges of patient safety programmes that focus on single outcomes within well-bounded healthcare settings that obscure individual’s experiences across pathways of care and exposure to multiple adverse events.	Broad: political, economic, social. Organisational context. External contextual influences—importance of ‘supportive outer context’ and how it can influence the impact of the collaborative approach.
Reed et al. 2018, UK [64]	72 Ohio maternity hospitals; 2 hospitals (Scotland and USA: 4 QI projects within each hospital)	Ohio: improve birth registry accuracy and reduce elective deliveries < 39 weeks. Scotland/USA: broad range of 8 QI projects set within two hospitals in Scotland and the USA.	Understand the influence of contextual factors in influencing QI & implementation (QI&I) initiatives within a broad range of settings—through the secondary analysis of qualitative data from two studies examining QI collaboratives/projects.	Dynamic with multiple, closely linked factors operating at different levels in a system that is constantly changing in response to QI&I initiatives. Three distinct types of context were identified: the setting(s) of care in which QI&I takes place; the context of the team conducting a specific project; the wider context supporting general QI&I.
Rostami et al. 2018, UK [65]	Medication safety in primary and secondary care	Implementation of a national Medication Safety Thermometer tool.	Reduction of medication-related harm is impeded by lack of routine medication safety data and standardised monitoring processes.	Organisational readiness, organisational culture, adaptation of intervention.
Rotteau et al. 2015, Canada [66]	Emergency departments	Reduce length of stay and improve patient flow.	Crowding in emergency departments is associated with poor patient experience, low staff morale and adverse patient outcomes. Examine how Lean can best be implanted in healthcare settings.	Structural, political, emotional, cultural.
Rycroft-Malone et al. 2013, UK [67]	Hospital wards providing pre/post-op care	Implementation of two evidenced-based guidelines about peri-operative fasting and resumption of fluids (3 intervention approaches were tested).	Gaps in literature around processes of implementation—using the issue around the variable evidence-base about the effectiveness of peri-operative fasting interventions—three trial implementation interventions were developed and randomly allocated—which included the prospective use of PARIHS .	Implementation context: micro (individual), meso (team), macro (hospital).
Schierhout et al. 2013, Australia [68]	Community-based health centres	Support best practice in prevention and management of chronic disease in indigenous primary health care services in Australia.	Improvement in quality of care for Indigenous Australians.	Regional and organisational infrastructure/culture.
Sommerbakk et al. 2016, Norway [69]	Two hospitals, one nursing home, two local medical centres (short-term inpatient care)	IMPACT (IMplementation of quality indicators in Palliative Care sTudy).	To meet the increased demand for palliative care (PC), efficient strategies are necessary to implement and/or improve PC at all levels of health care, not just in specialist settings.	Social (e.g. leadership, culture of change, face-to-face contact); organisational (e.g. resources, structures/facilities, expertise); political and economic (e.g. policy, legislation, financial arrangements).
Sutton et al. 2016, UK [70]	Transitions of care across care boundaries— between residential care settings and hospital	Reduce unplanned readmissions from residential care homes.	Suboptimal transitional care between hospitals and residential care settings—addressing continuity and coordination issues.	Inter-organisational.
Tomoaia-Cotisel et al. 2013, USA [71]	Primary care practices	Transform primary care practices into patient-centred medical homes.	Transformation of primary care services to improve outcomes and processes. Translating research into practice often fails due to lack of knowledge around contextual factors and how they modify intervention effects.	Practice setting, larger organisation, external environment, implementation pathway, motivation for implementation.

Secondary care was the most predominant setting (*n* = 22) and the majority of the studies reported macro-level results from across more than one hospital or organisation. The studies involved participants with a wide range of experience, including clinicians, organisation leaders, managers, support staff and internal/external QI facilitators, from a variety of settings. Most of the studies reported on improvement initiatives targeting specific systems or processes, such as the implementation of preventive care practices, care bundles, patient safety practices, evidence-based guidelines, checklists or the redesign of clinical pathways/processes. Other types of QI activities included continuous quality improvement (CQI). A variety of standard QI methods and tools were described across the studies. Eleven studies reported on QI collaborative models. Reporting on QI methodology in a small number of the studies was of poor quality, with a lack of detail on the specific improvement methods used.

### Main findings

In this section, the various representations of context across the included studies are first explored. Then, we describe the four key domains that emerged from the data—leadership, organisational characteristics, change agents and multi-disciplinary collaboration—reflecting contextual influences at levels of the system. Findings from the evidence synthesis further distilled the four domains into eight key contextual factors: leadership, organisational culture, individual skills and capabilities, organisational capacity and capability, data and technical infrastructure, readiness for change, championship and relationships. The contextual factors were shown to interact across healthcare system levels (macro, meso and micro), during the stages of improvement. A generalisable theoretical model was then developed to illustrate the interactions between contextual factors, system levels and the various stages of the improvement journey along a trajectory where improvements are planned, implemented, sustained and spread.

#### Representations of context within improvement settings

Within the studies reviewed, context was represented in a variety of ways within the literature, highlighting its dynamic, multi-dimensional and highly variable nature. It was characterised both within and across studies as political, economic, social, inner/outer setting, institutional, organisational and individual. However, context was also strongly intertwined with ‘culture’, both at local and organisational levels [40–44, 46, 52, 53, 55–57, 62, 65, 66]. It was also used as a means to demonstrate system complexity, through the interactions at the micro, meso and macro system levels [38, 44, 46, 47, 49, 54, 57, 64, 67, 68, 71], supporting the programme theory. Multiple interactions between different aspects of context were reported across the evidence, for example the influence of macro-level contexts on the micro system or the tensions and trade-offs between the two [38, 43, 49, 52, 53, 57, 60, 63].

Some studies attempted to define context within a hierarchy of factors [47, 54, 58, 71]. Others made a clear distinction between local contexts (as the implementation setting) and the broader contextual landscape [43]; for example, one study identified three distinct types of context—the setting of care context, the project-specific context and the wider general QI and implementation (QI&I) context [64]. Many studies considered and compared pre- and post-implementation contexts [38, 41, 46, 48, 67].

Awareness of the potential impact of implementation contexts and local conditions featured widely in the literature in a range of forms. Context was described both in negative and positive terms: frequently contextual factors were portrayed as barriers and/or facilitators [46–48, 50, 69], and in one instance, context was viewed as a continuum of conditions from positive/favourable/strong influences to negative/unfavourable/weak influences [58]. Interrelationships among contextual elements acted as barriers to uptake at some sites and as facilitators at other sites, and as such were a predictor of intervention uptake [58]. Some studies explored implementation in multiple settings, highlighting that conditions for readiness, underlying mechanisms and outcomes of the same intervention could be very different depending on the organisational context [43, 52, 53, 56, 60, 66].

#### Context assessment

A ‘context assessment’ process [51, 71] was reported in a number of studies, often synonymous with pre-implementation quality planning, and preparation for implementation, spread and sustainability prior to the start of a project. These assessments aimed to build an in-depth understanding of the setting (internal context), tasks, outcomes and environment into which the initiative would be introduced. Assessments included the examination of organisational structures and processes, i.e. pre-existing patterns of working and communication mechanisms. In practical terms, this involved actively engaging frontline staff and other stakeholders and encouraging participation or co-design [39, 43, 45, 46, 54], assessing and/or developing capacity and capability in clinical microsystems [42, 46, 52, 57] and addressing organisational readiness prior to implementation [54, 66].

In some studies, teams utilised QI methods as tools to help them understand and analyse the complexity of their systems [59], whereas others used specific frameworks to systematically evaluate their local context and identify relevant contextual factors to address, e.g. the context curriculum developed using MUSIQ, which facilitated teams’ use of QI methodology to address contextual factors to reduce barriers and support implementation [51].

#### Managing context

In contrast to the design of ‘bespoke’ programmes developed for individual contexts (i.e. ‘contextualised’ to specific settings), a process of tailoring to context or ‘adapting’ to achieve fit was described [56]. This acknowledges activities often undertaken to modify improvement initiatives and implementation approaches to suit local conditions, in order to achieve ‘fit’ or integration with existing practices. Interactions between context and intervention were reported [54, 68] and the inter-relationship between intervention and context to achieve fit worked both ways: other studies reported on attempts to modify or ‘improve’ the local context prior to QI implementation in order to make it more supportive/receptive or to ameliorate the effects of contextual factors that impeded improvement efforts [48, 51]. An example commonly mentioned was the alignment of national priorities to local work/improvement contexts or vice-versa [49, 53, 63]. However, misalignment of QI programme goals to local conditions/priorities, or an inability to achieve fit, was also highlighted [37, 38, 43, 61, 70] as a challenge when a mismatch between features of programme design/delivery and implementation contexts occurred.

### Data domains

Using the programme theory (Fig. 3) as a framework, the 35 review studies were examined to identify which contextual factors influenced the implementation, effectiveness, sustainability and transferability of QI initiatives within healthcare. Analysing the data against the programme theory, four key domains emerged—*leadership, organisational characteristics, change agents* and *multi-disciplinary collaboration.*

### Leadership

Leadership was a key element in the review programme theory—both within the ‘organisation’ context and as a mechanism to deliver outcomes.

### Organisational characteristics

Supporting ‘organisation’ as a key context in the programme theory, this domain emerged very strongly from the evidence, broadly reflecting system-level (micro/meso) and individual-level contextual factors. It included organisational structures, processes and human resource functions. Awareness of the potential impact of organisational contexts and local conditions are featured widely in the review and included examples of context assessment. The evidence showed that organisational infrastructure should ideally be supportive of improvement, and a key element identified was organisational ‘culture’, i.e. the history, policies, strategy and governance of an organisation, underpinned by shared vision, beliefs and patterns of behaviour. These core values, attitudes, norms and underlying ideologies shaped the implementation context in multiple ways, alongside the level of organisational ‘QI maturity’. An organisation’s QI maturity was highlighted in a number of studies as being one of the key factors influencing the successful implementation of an improvement initiative.

### Change agents

Consistent with the programme theory descriptions of the various roles of individuals as ‘drivers of QI’, leading change within the key ‘stakeholder’ context, the role of change agents was frequently reported in the evidence in a variety of forms, from local champions to external QI experts.

### Multi-disciplinary collaboration

Multi-disciplinary collaboration featured very strongly across the included studies, despite playing a lesser role in the programme theory, where it was conceptualised as both mechanism and outcome. Interconnected elements within this domain included professional diversity, relationship building, teamwork and communication; these reinforced other aspects of the programme theory.

These four domains reflected contextual influences at all levels of the system. Examples from the realist exploration of *how*, *why*, *when* and *for whom* these contextual domains are important to improvement initiatives are provided in Table 3.

**Table 3:** Contextual domains

Domain	How?	Why?	When?	For whom?
Leadership	Leaders enthusiastic about change had a motivational effect on staff, and the involvement of leaders or executive sponsors lead to a more positive experience of improvement.	Encouraging leadership leads to shared responsibility and increased accountability—promoting progress and staff empowerment.	To support motivational efforts and address operational and organisational barriers.	Professional groups and frontline staff.
Organisational characteristics	Empowers individuals/teams to achieve change. Formal structures (data, training) influence how an organisation delivers improvement initiatives.	The QI infrastructure reflects an organisation’s ability to intentionally, and systematically use improvement approaches to change processes and improve outcomes. It demonstrates an organisation’s readiness for change.	Planning and pre-implementation phases—prior to the adoption or spread of an improvement initiative.	Individuals and frontline teams.
Change agents	Drive initiatives, enable others and/or hold teams accountable for implementation.	Facilitate engagement with stakeholders, and help teams identify problems, develop solutions and understand the use QI methodologies.	All stages in the improvement journey.	A wide range of staff, including individuals and frontline teams.
Multi-disciplinary collaboration	Fosters team ownership and shared goals across the organisational system levels.	Flattens hierarchies and aligns the interests of multiple stakeholders.	All stages in the improvement journey.	Actors at various organisational and system levels.

### Mechanisms

A number of key mechanisms that influenced the delivery of quality improvement initiatives (outcomes) were identified from the literature, supporting the programme theory. The mechanisms were applied to different system levels (Table 4).

**Table 4:** Mechanisms

Initial programme theory mechanism	Refined programme theory mechanism	Response triggered by intervention	System level influence
Creating the Culture	Empowerment	Staff with autonomy to initiate improvement and come up with ideas/solutions; increases their desire to become involved.	Organisational structures to support autonomy.
	Ownership	Ownership of QI drives improvement activities.	Micro level—operational context (ward/clinic) where change takes place. Macro/meso level—organisational ownership, engaging with national initiatives and being able to translate them to local priorities.
Frontline engagement	Engagement	Engagement with QI efforts fostered by interest, active involvement and autonomy.	Needs micro/meso/macro level commitment.
Informed practitioners	QI capability building	Micro level—empowers frontline staff to lead initiative and increased confidence show/tell other staff.	Needs micro/meso/macro level commitment.
	QI capacity building	Micro level—enabling via the provision of resources and support: building skills, knowledge, relationships and the confidence to enact change.	Needs micro/meso/macro level commitment.
Strong leadership	Psychological ‘safety’	Micro level—freedom to voice concerns; characterised by openness, trust and open communication.	Organisational macro/meso structures to facilitate that psychological safety within QI work.
	Motivation	Micro level—motivation of staff.	Macro/meso level support for improvement.

### Context mapping

**Fig. 4:**
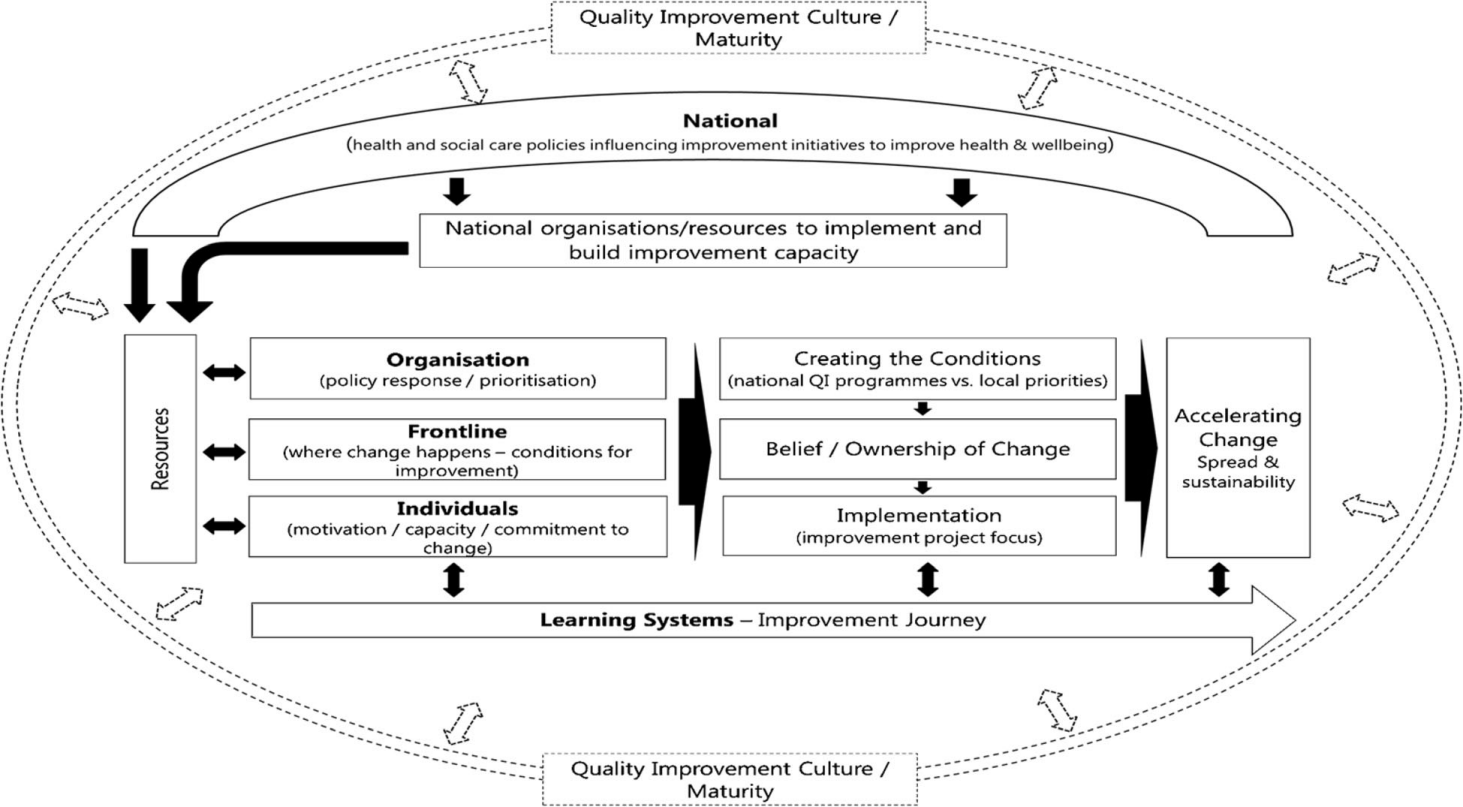
Revised context map

The map of the theoretical and conceptual landscape of healthcare QI was redrawn with the integration of tacit knowledge from stakeholders, to produce a broader, more descriptive model (Fig. 4), refining the refined interpretations of context that had emerged from review findings. Given the interpretive and subjective nature of the realist approach [72, 73], this sense-checking exercise was invaluable. Revising the context map enabled progression beyond the data domains, towards an enhanced understanding and the identification of contextual factors and their influence and impact.

### Contextual factors

Findings from the evidence synthesis further distilled the four domains into eight key contextual factors (Table 5): *leadership*, *organisational culture*, *individual skills and capabilities*, *organisational capacity and capability*, *data and technical infrastructure*, *readiness for change*, *championship and relationships*.

**Table 5:** Contextual factors

Domain	Factor	Description
Leadership	Leadership	Supportive, active, engaged, effective, consistent, motivational, accessible, credible. Belief in QI. Blended leadership approach (top-down/bottom-up).
Organisational characteristics	Organisational culture	Core values, attitudes, norms, systems, processes. Underlying ethos and principles. History. Implementation climate. Organisational commitment.
	Individual skills and capabilities	Individuals and groups/teams: QI expertise, understanding prior experience. Training, learning, development of a skill set to address ‘QI skills gap’.
	Organisational capacity and capability	Improvement culture, prior initiatives, QI history and maturity. QI capacity. Developing or ongoing ‘organisational learning’. Availability of dedicated resources.
	Data and technical infrastructure	Systems, measurement, monitoring, feedback: availability and use of data as a motivator to improve. Information systems in place to support systematic and standardised collection and use of data for improvement. Integration of data collection into existing practices to minimise ‘burden’ on staff. Technical capability of staff to use data.
	Readiness for change	Receptiveness, shared resolve, belief, support, commitment, collective change efficacy.
Change agents	Championship	Change agents: driving and leading change. Ownership, engagement, participation.
Multi-disciplinary collaboration	Relationships	Collaboration: multidisciplinary, formal/informal, external. Strong working relationships. Facilitation of communication across all levels. Support for networks. Consensus-building.

### Explanatory theoretical model

**Fig. 5:**
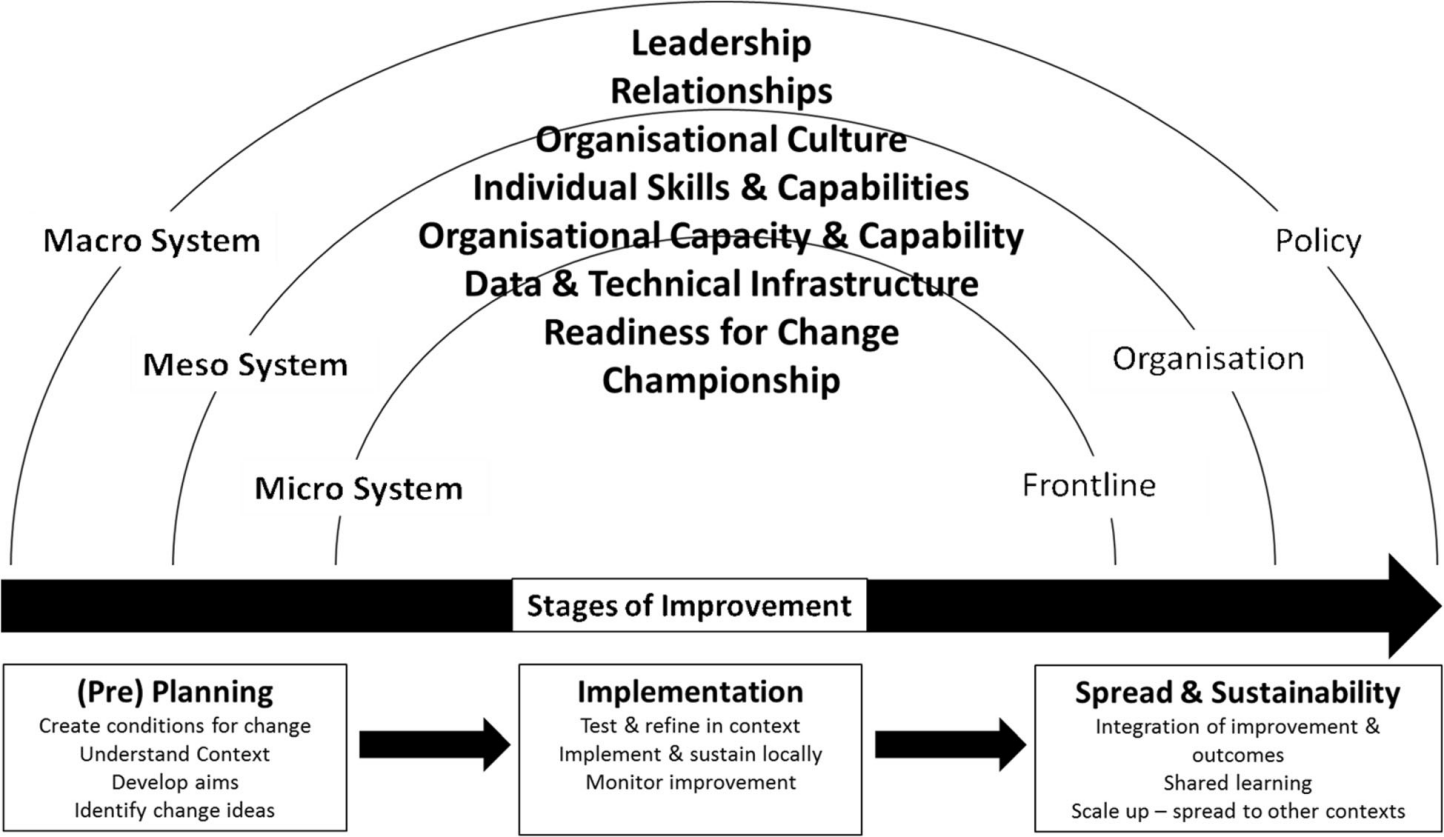
Evidence-informed explanatory theoretical model

A generalisable theoretical model (Fig. 5) was developed to illustrate the interactions between contextual factors, system levels and the various stages of the improvement journey along a trajectory where improvements are planned, implemented, sustained and spread.

Recognising that context runs all the way through the improvement continuum, we anticipate that the model could support the examination of contextual factors’ influence at each system level during the stages of an improvement initiative. From pre-planning onwards, this model could enhance the understanding of the QI context, and the dynamic, complex systems within it, whilst acknowledging the variation of contextual factors between settings.

## Discussion

We identified four contextual domains that influence the implementation, effectiveness, sustainability and transferability of QI initiatives in healthcare. Further unpacking of these domains led to the distillation of eight key contextual factors, which apply to multiple system levels in varied healthcare settings. This review demonstrates the impact of context across all stages of the improvement journey, from the pre-planning and implementation stages, towards achieving spread and sustainability. Our findings follow Øvretveit et al.’s [74] view of contextual conditions as ‘influences which interact with each other, and interact with the implementation process’; however, we also acknowledge the dynamic relationship between contextual factors and system levels that occurs during the entire cycle of improvement, pre- and post-implementation. The mutually emergent domains and contextual factors identified in this review are all interconnected—and to a lesser degree, overlapping—so they cannot be viewed in isolation, underlining the complex systems approach to QI [3, 24]. What also became apparent during the course of the review is that the key mechanisms underlying successful quality improvement initiatives are relational and social processes that are fluid, flexible and interrelated (Table 4), played out within equally fluctuating contexts and constantly changing systems. These processes do not easily fit into a structured model.

Within realist reviews, it is common practice to refine the programme theory and CMO configurations (context + mechanism = outcome). The outcomes for this review are reflected as the eight contextual factors within the evidence-informed theoretical explanatory model (Fig. 5), rather than specific standalone outcomes within CMO configurations. The contexts, mechanisms and outcomes interrelate and overlap between the system levels and their influence at those levels change, along with the role they play within the CMO configurations. For example:

**Table:** 

Context	Mechanism	Outcome
National: government policies	Consistent support for QI through policies and strategies	Leadership within organisations
Organisation: leadership	Organisational response	Organisational capacity and capability
Clinical microsystem: individuals	Developing capacity for improvement	Individual skills and capabilities

The original review programme theory was formed around four main contexts: the national policy context (macro); the organisational context (including leadership, culture and systems—macro/meso); the clinical microsystem (where most improvements take place—meso/micro); and the stakeholder context (conceptualised as the context of ‘individual change’—micro).

Review findings strongly identified with the latter three contexts and explored the context in and around interventions at these levels, emphasising the dimensions within the organisational context, with a focus on system levels, reinforcing the notion that most improvement and change is taking place at the micro and meso levels. However, whilst that was the primary focus for the majority of studies, the national or macro context was commonly seen as an overarching contextual influence on QI initiatives. Studies frequently reported that regional or national government policies or national agency directives were behind the initiatives. This illustrates the external contextual influences on organisations and the importance of the organisational characteristics, as key mechanisms working to align and accept the ‘change’ and support the initiative.

‘Organisational characteristics’ was identified as one of the four contextual domains, and context assessments were highlighted in the review as a mechanism to gain a greater understanding of internal organisational contextual influences and readiness for change. The key elements within this domain are related to culture and the multiple subcultures that can act both to drive change and to undermine improvement efforts [75]. Lukas et al. [76] suggest that in some instances the clinical microsystem culture may assume more significance to improvement than the wider organisational culture, which ties in with the importance of clinical microsystems within the review programme theory. The challenges of organisational contexts, ranging from individual/team capacity and capability to deliver QI, to the infrastructures that support an organisation’s learning and technical capacity to plan, manage and monitor improvements, have also been noted elsewhere [75, 77–80].

Across the review evidence, there was a strong focus on the role of leadership in various forms. Within the programme theory, leadership was originally characterised as ‘strong leadership’; this was later revised in light of discussions with stakeholders. They viewed successful leaders as ‘active’ and ‘supportive’ within a high-trust environment where all staff are encouraged to lead improvement, reinforcing the notion of a blended leadership model at different levels of the system—demonstrated in a number of the review studies. Also highlighted was the close interaction between leadership and organisational culture, which in turn influenced improvement leadership and management.

This review emphasises the importance of stakeholders (individuals/teams and their capacities and skills) within the improvement process, particularly around the key roles of change agents and champions [19, 81] in supporting the success of QI interventions at the microsystem and senior organisational support for initiatives. The ‘change agents’ domain and contextual factor ‘championship’ align with McCormack et al.’s [81] conceptualisation of change agency as ‘roles that are aimed at effecting successful change in individuals and organizations’.

### Review strengths and limitations

The use of a realist approach in this review was a key strength. Input from stakeholders with practical knowledge and experience of quality improvement within healthcare settings facilitated the generation of conceptually rich findings with theoretical depth. Stakeholder input and feedback were incorporated at various stages of the review process, playing an important role in generating the initial CMO configurations and to ‘sense check’ and refine the theoretical framework and the final outputs. This contribution helped to temper the interpretive and potentially subjective nature of realist synthesis and provided validation, adding credibility and grounding the findings in real-world knowledge and experience.

A limitation of this review is that a small number of studies were weak in terms of their descriptions of QI methodology and lacked detail on the specific improvement methods or tools used within the study. In a number of studies, the assessment of context was conducted retrospectively rather than prospectively, which is less likely to produce meaningful, transferable findings and avoid recall bias [82]. However, despite this limitation, most studies included (either explicit or implicit) rich contextual information, which contributed to the review.

Further, it must be noted that the included literature was predominantly from the acute care sector (two-thirds of the studies were in hospital settings), based in the USA and UK. Other reviews have demonstrated similarities [10, 23, 34, 78], which is unsurprising, given that the origins of QI in healthcare can be traced to the USA [1] and most QI training curricula are founded in the USA [83]. For the purposes of this review, the focus was higher-income countries, where QI approaches tend to be more embedded and ‘mature’ due to the established emphasis on improving the quality of healthcare systems through the use of QI methodologies.

### Implications for future research and practice

The explanatory theoretical model provides a practical reflection of the current evidence base in relation to the influence of context on improvement activities. As an ‘aide memoir’, it can encourage the contemplation of contextual factors by practitioners, senior staff and policy makers to enhance the delivery of improvement initiatives. The next stage of this work will be to maximise the learning from the study, and to consider the application of the explanatory theoretical model in real-world settings: working with researchers and improvement practitioners, to facilitate the translation of the review findings and theoretical model into practical application. This will involve the consideration and co-development of *who* will use it (e.g. clinical teams, QI practitioners, organisations), *when* it should be used (e.g. at the pre-planning stage of local initiatives, planning the adoption of national initiatives, or to spread existing improvements to other contexts) and *what* form it should take. This co-development process will enable practitioners to have a greater understanding of the influence of context on quality improvement initiatives and will give researchers an opportunity to evaluate the impact of a more context-sensitive approach to the design and implementation of improvement initiatives, across health and social care, to advance practice and accelerate change.

## Conclusion

To our knowledge, this is the first realist review investigating the influence of context in healthcare quality improvement. The use of a realist approach enabled identification of the key contextual factors that influence QI initiatives in healthcare and provides a theoretical explanation of how, why, when and for whom these contextual factors are important to QI initiatives, at different system levels and during the stages of improvement. This review enhances the evidence base around context in QI and addresses the limited knowledge and guidance about which contextual factors to consider.

Our explanatory theoretical model reflects that the interplay between improvement interventions and their context is a fluid interaction and as such, each can influence the other directly and indirectly in multiple ways. Working within complex adaptive systems, the model reflects the current evidence base around context and provides practitioners with an informed approach to consider how the influence of these contextual factors will impact within their own setting. The factors are not weighted by importance, as their influence will vary from setting to setting. The model provides a practical ‘aide memoir’ that supports pre-planning conversations at the micro system level, either at the start of an initiative or more importantly when spreading changes from one setting to another that can be applied to multiple settings that are constant state of change.

This research has produced the foundations to enhance improvement practices, through the co-development with improvement practitioners and policy makers to advance knowledge and the practical assessment of the role and influence of context in healthcare practice settings.

## Data Availability

The datasets generated and analysed during this study are available from the corresponding author on reasonable request.

## Abbreviations

CMO: Context-mechanism-outcome
MUSIQ: Model for understanding success in quality
PARIHS: Promoting Action on Research Implementation in Health Services
QI: Quality improvement
RAMESES: Realist And Meta-narrative Evidence Syntheses: Evolving Standards
SQUIRE: Standards for QUality Improvement Reporting Excellence
